# Vascular compactness of unruptured brain arteriovenous malformation predicts risk of hemorrhage after stereotactic radiosurgery

**DOI:** 10.1038/s41598-024-54369-2

**Published:** 2024-02-18

**Authors:** Po-Wei Huang, Syu-Jyun Peng, David Hung-Chi Pan, Huai-Che Yang, Jo-Ting Tsai, Cheng-Ying Shiau, I-Chang Su, Ching-Jen Chen, Hsiu-Mei Wu, Chung-Jung Lin, Wen-Yuh Chung, Wan-Yuo Guo, Wei-Lun Lo, Shao-Wen Lai, Cheng-Chia Lee

**Affiliations:** 1 Department of Radiation Oncology, Shuang Ho Hospital, Taipei Medical University, New Taipei City, Taiwan; 2 Program in Artificial Intelligence in Medicine, College of Medicine, Taipei Medical University, Taipei, Taiwan; 3 Department of Neurosurgery, Neurological Institute, Taipei Veterans General Hospital, Taipei, Taiwan; 4 Department of Neurosurgery, Shuang Ho Hospital, Taipei Medical University, New Taipei City, Taiwan; 5 Department of Neurosurgery, University of Texas Health Science Center, Houston, TX USA; 6 Department of Radiology, Taipei Veterans General Hospital, Taipei, Taiwan; 7 Cancer Center, Taipei Veterans General Hospital, Taipei, Taiwan; 8 School of Medicine, National Yang Ming Chiao Tung University, Taipei, Taiwan; 9 Brain Research Center, National Yang Ming Chiao Tung University, Taipei, Taiwan; 10 Department of Radiology, School of Medicine, College of Medicine, Taipei Medical University, Taipei, Taiwan; 11 Department of Surgery, School of Medicine, College of Medicine, Taipei Medical University, Taipei, Taiwan; 12 Graduate Institute of Clinical Medicine, College of Medicine, Taipei Medical University, Taipei, Taiwan; 13 Taipei Neuroscience Institute, Taipei Medical University, Taipei, Taiwan; 14 Product and Engineering, Zippin, San Carlos, CA USA

**Keywords:** Stereotactic radiosurgery, Post-SRS hemorrhage, Compactness index, Automated segmentation, Arteriovenous malformation morphology, Vascular disorders, Neurological disorders, Mathematics and computing, Outcomes research

## Abstract

The aim of the study was to investigate whether morphology (i.e. compact/diffuse) of brain arteriovenous malformations (bAVMs) correlates with the incidence of hemorrhagic events in patients receiving Stereotactic Radiosurgery (SRS) for unruptured bAVMs. This retrospective study included 262 adult patients with unruptured bAVMs who underwent upfront SRS. Hemorrhagic events were defined as evidence of blood on CT or MRI. The morphology of bAVMs was evaluated using automated segmentation which calculated the proportion of vessel, brain tissue, and cerebrospinal fluid in bAVMs on T2-weighted MRI. Compactness index, defined as the ratio of vessel to brain tissue, categorized bAVMs into compact and diffuse types based on the optimal cutoff. Cox proportional hazard model was used to identify the independent factors for post-SRS hemorrhage. The median clinical follow-ups was 62.1 months. Post-SRS hemorrhage occurred in 13 (5.0%) patients and one of them had two bleeds, resulting in an annual bleeding rate of 0.8%. Multivariable analysis revealed bAVM morphology (compact versus diffuse), bAVM volume, and prescribed margin dose were significant predictors. The post-SRS hemorrhage rate increased with larger bAVM volume only among the diffuse nidi (1.7 versus 14.9 versus 30.6 hemorrhage per 1000 person-years in bAVM volume < 20 cm^3^ versus 20–40 cm^3^ versus > 40 cm^3^; p = 0.022). The significantly higher post-SRS hemorrhage rate of Spetzler-Martin grade IV–V compared with grade I–III bAVMs (20.0 versus 3.3 hemorrhages per 1000 person-years; p = 0.001) mainly originated from the diffuse bAVMs rather than the compact subgroup (30.9 versus 4.8 hemorrhages per 1000 person-years; p = 0.035). Compact and smaller bAVMs, with higher prescribed margin dose harbor lower risks of post-SRS hemorrhage. The post-SRS hemorrhage rate exceeded 2.2% annually within the diffuse and large (> 40 cm^3^) bAVMs and the diffuse Spetzler-Martin IV–V bAVMs. These findings may help guide patient selection of SRS for the unruptured bAVMs.

## Introduction

Brain arteriovenous malformations (bAVMs) harbor an annual bleeding risk ranging between 2 and 4%^1–4^. The neurological morbidity or mortality following bAVM rupture upholds intervention. The goal of the bAVM treatment is to eliminate or reduce the annual bleeding risk. However, for the unruptured bAVMs, the potential benefit of preventive treatment should be cautiously weighed against the lower natural bleeding risk (1% to 3%)^4–6^.

Previous studies have reported various predictors for bAVM hemorrhage after SRS. Karlsson et al. revealed that patient age, bAVM volume, and minimum dose were independent predictors^7,8^. Patibandla et al. demonstrated a significant association between bAVM volume and post-radiosurgery hemorrhage in Spetzler-Martin IV–V bAVMs^9^. Furthermore, Ding et al. observed a higher hemorrhage rate in the Spetzler-Martin grade IV–V bAVMs after SRS compared to the pre-treatment estimate^10^. These findings, however not exclusively the unruptured presentation, shed light on the importance of risk stratification and patient selection for bAVM management with SRS.

Despite comprehensive studies on bAVM hemorrhage after SRS, the effect of bAVM morphology (compact or diffuse) is infrequently discussed. Previous studies have developed and validated an automated segmentation algorithm to differentiate vessel, brain and CSF components of bAVMs^11,12^. Compactness index, defined as the ratio of the vasculature proportion to the brain tissue proportion, was utilized to quantify the bAVM compactness^13^. In this study, we applied the algorithm and formula to investigate the impact of bAVM compactness on the occurrence of post-SRS hemorrhage in patients with unruptured bAVMs.

## Results

### Patient, bAVM characteristics and automated segmentation of bAVMs

The study included 262 patients with unruptured bAVMs. The patient demographics, bAVM characteristics, dosimetry parameters, and automated segmentation results are listed in Table 1. The median age of the patients was 36.0 years. The median (range) bAVM volume was 17.6 (1.4–71.6) cm^3^. The mean bAVM volume was 22.3 cm^3^. The 1st and 3rd quartiles were 10.6 and 31.8 cm^3^. The median margin dose and target mean dose were 17.5 Gy and 22.7 Gy. The median (range) of 12Gy irradiated volume was 32.5 (2.8–111.3) cm^3^. The median (range) image and clinical follow-up duration were 42.9 (5.3–185.0) months and 61.8 (5.3–240.6) months. Of note, 136 patients had follow-up more than 5 years, and 56 patients had more than 10 years of follow-up.

**Table 1:** Summary of patient, bAVM and SRS characteristics.

Characteristics	Value
Age (median [range] years)	36.0 [18.9–80.1]
Sex (%)	
Male	143 (54.6)
Female	119 (45.4)
Presentation (%)	
Seizure	129 (49.2)
Neurologic deficit	58 (22.1)
Headache	51 (19.5)
Asymptomatic	24 (9.2)
bAVM location (%)	
Cerebral hemisphere	222 (84.7)
Thalamus, basal ganglia, brainstem	18 (6.9)
Cerebellum	10 (3.8)
Corpus callosum	9 (3.4)
Intraventricular	3 (1.1)
Aneurysm (%)	
Intranidal and/or proximal	28 (10.7)
Venous/unrelated	18 (6.9)
bAVM volume (median [range] cm^3^)	17.6 [1.4–71.6]
Eloquent AVM location (%)	176 (67.2)
Deep venous drainage (%)	107 (40.8)
Spetzler-Martin grade (%)	
I	33 (12.6)
II	75 (28.6)
III	86 (32.8)
IV	53 (20.2)
V	15 (5.7)
Radiosurgery-based score (%)	
< 1.00	8 (3.1)
1.00–1.50	32 (12.2)
1.50–2.00	33 (12.6)
> 2.00	189 (72.1)
Radiosurgery-based score (median [range])	2.6 [0.6–8.5]
Virginia radiosurgery AVM scale (%)	
0	2 (0.8)
1	14 (5.3)
2	94 (35.9)
3	152 (58.0)
4	0 (0)
Repeated treatment for remnant bAVM (%)	36 (13.7)
GKRS	34 (13.0)
Embolization	4 (1.5)
Image follow-up duration (median [range] months)	42.9 [5.3–185.0]
Clinical follow-up duration (median [range] months)	61.8 [5.3–240.6]

Dosimetry	Value
Margin dose (median [range] Gy)	17.5 [14.5–20.0]
Maximum dose (median [range] Gy)	30.5 [25.9–37.3]
Target mean dose (median [range] Gy)	22.7 [18.2–26.1]
Isodose level (median [range] %)	57.0 [50.0–65.0]
Gradient index (median [range])	2.9 [1.8–3.8]
Target coverage (median [range] %)	95.2 [85.6–99.8]

Automated segmentation of bAVM	Value
Vascular proportion (median [range] %)	30.9 [13.5–66.1]
Brain parenchyma proportion (median [range] %)	52.2 [17.6–73.7]
CSF proportion (median [range] %)	16.8 [6.9–41.9]
Compactness index (median [range])	0.6 [0.2–3.7]

**Figure 1:**
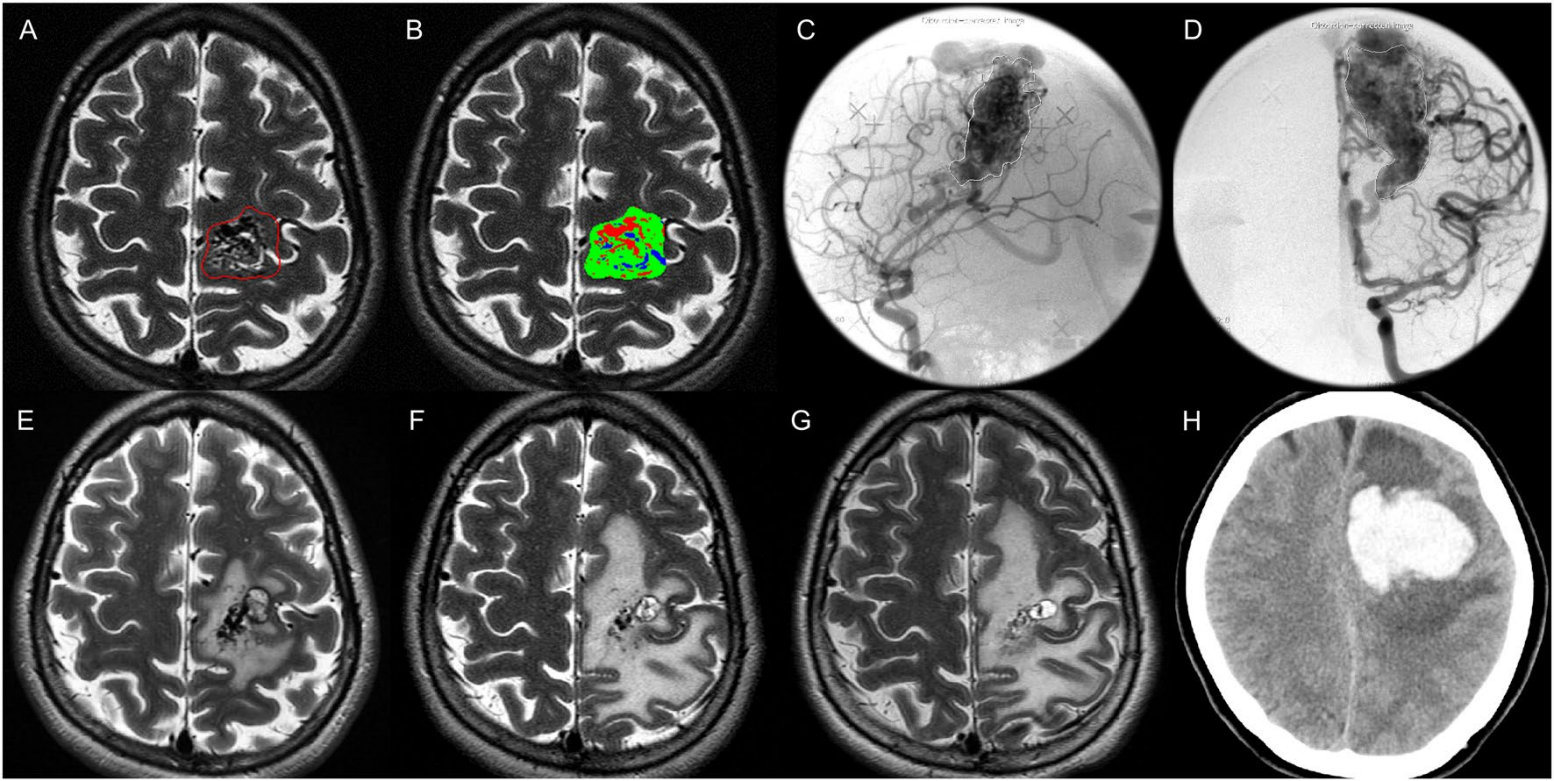
A case demonstration of fully automated segmentation results and follow-up images. This 30-year-old female with a left frontal lobe bAVM presented as neurological deficit. The bAVM volume and the prescribed margin dose were 24.4 cm^3^ and 17.2 Gy. (**A**, **B**) The automated segmentation results within the prescription isodose volume. (*Red* indicates the vascular component, *green* indicates the brain parenchyma, and *blue* indicates the CSF). The compactness index was 0.43, which allocated the bAVM into diffuse morphological type. (**C**, **D**) Lateral and anteroposterior angiography of the left internal carotid artery. (**E**–**G**) The follow-up MRI at 8, 15 and 21 months after SRS showed a residual nidus. (**H**) The patient suffered a hemorrhage event 22 months after SRS as demonstrated on the non-contrast CT.

The automated segmentation algorithm differentiated the constituents in each bAVM. The proportions of vessel, brain parenchyma, and CSF were 30.9%, 52.2%, and 16.8%. The median (range) compactness index was 0.58 (0.24–3.66). The optimal cutoff of the compactness index was 0.63, which was determined by the ROC curve and Youden indices. 111 (42.4%) unruptured bAVMs with compactness index greater than 0.63 were categorized into compact bAVMs, while the remaining 151 (57.6%) were classified as diffuse bAVMs. Figure 1 demonstrates the segmentation result of a diffuse bAVM (compactness index 0.43) and the follow-up MR images after SRS. Another case demonstration is presented in Supplementary Fig. 1.

### Complete obliteration and RICs

195 (74.4%) patients achieved complete obliteration. Of these patients, 141 (72.3%) were confirmed with DSA and 54 (27.7%) were confirmed with only MRI. The actuarial CO rates were 55.8%, 66.0%, 72.9%, and 80.9% at 3, 4, 5, and 6 years, respectively. Radiologic RICs occurred in 75.2% of patients. Grade I RICs were observed in 57.6% of patients, Grade II RICs in 15.6%, and Grade III RICs in 1.9% of patients. Permanent RICs were observed in 12 (4.6%) patients, manifesting as drug-resistant seizure, hemiparesis, memory impairment, visual field defect and hemiballism.

### Hemorrhage after SRS

12 patients had one hemorrhage event and 1 patient had two hemorrhage events. A total of 14 hemorrhagic events occurred in 1718 person-years, resulting in an annual bleeding rate of 0.8%. Supplementary Table 1 demonstrates the clinical details of each hemorrhagic event after SRS. We observed 1 fatal hemorrhage and 8 debilitating events with permanent neurological sequelae, while the deficits of the other 5 hemorrhagic events were temporary.

The median (range) time to hemorrhage after SRS was 37.4 (5.2–185.7) months. After SRS, 2 hemorrhages were observed in the first year, 3 in the second, and 2 in the third year. 7 hemorrhagic events occurred more than 3 years after SRS. The annual hemorrhage rate in the first 3 years and thereafter was 9.5 and 7.1 per 1000 person-years, respectively. If we censored the follow-up on the CO date for the obliterated bAVMs and on the latest follow-up for the non-obliterated nidi, the annual bleeding rate in the latency period was 13.6 per 1000 person-years. The rate in the first 3-year latency period and thereafter would be 10.3 and 19.6 per 1000 person-years.

**Figure 2:**
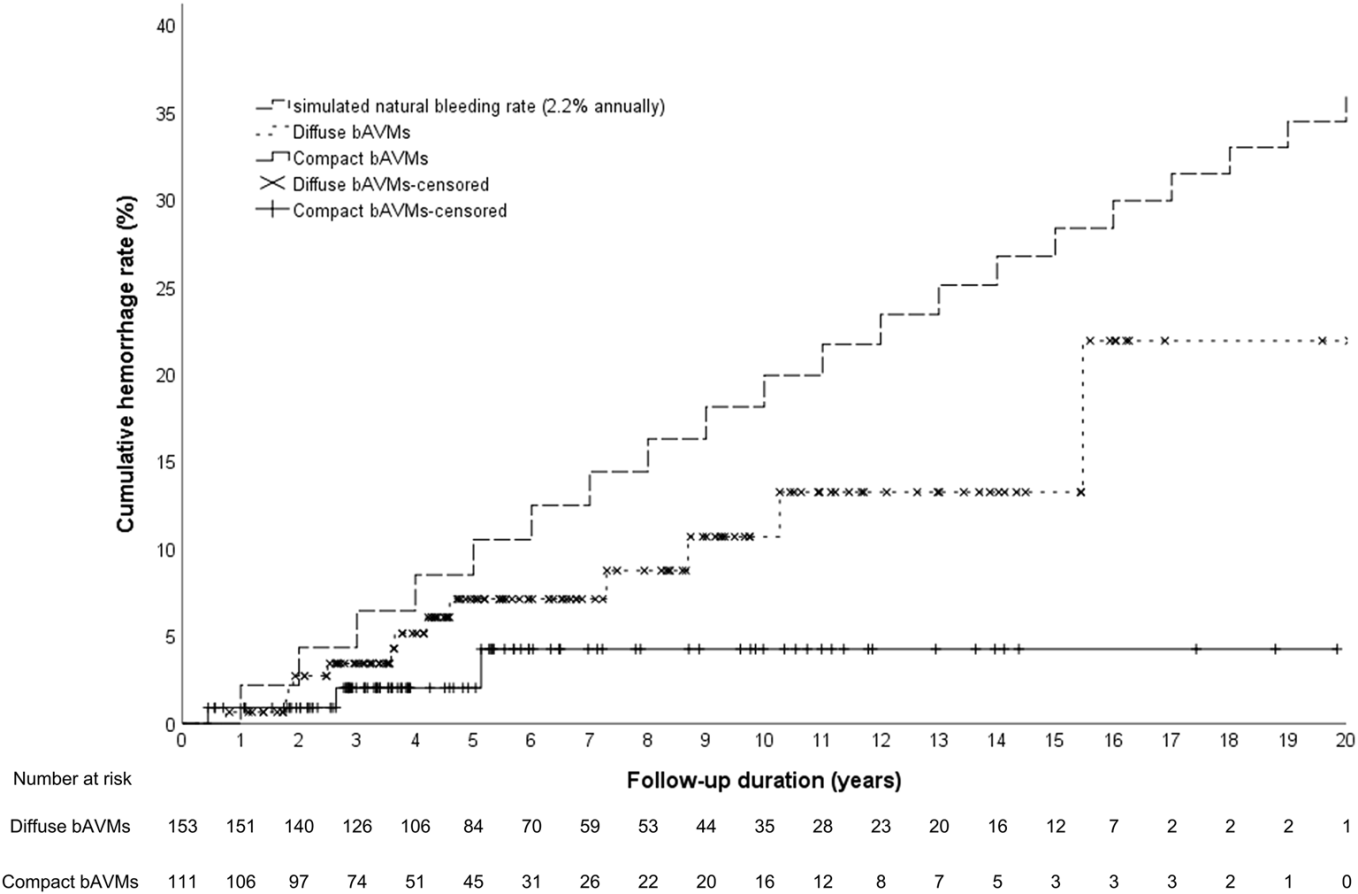
Kaplan–Meier plots of the simulated natural bleeding rate and the post-SRS hemorrhage rate over time among the compact, diffuse unruptured bAVMs. The post-SRS hemorrhage rate of both the diffuse and compact bAVMs were significantly lower than the natural course. (diffuse bAVMs versus natural course, log-rank test, p = 0.012; compact bAVMs versus natural course, log-rank test, p = 0.022).

One patient had two bleeds and was treated as two patients with post-SRS hemorrhage to reflect the doubled risk with certain factors in time-to-event analysis. The 1-, 3-, 5-, and 7-year actuarial hemorrhage rates were 2.0%, 4.0%, 5.5% and 6.7%. Kaplan–Meier analysis revealed that the hemorrhage rate of both the diffuse and compact bAVMs after SRS were significantly lower than the simulated natural cumulative bleeding rate with 2.2% annually (Fig. 2).

### Clinical factors, angioarchitecture associated with hemorrhage after SRS

In univariate analysis, bAVM volume, margin dose and target coverage were associated with post-SRS hemorrhage. In the multivariable analysis, compact bAVM (HR 0.25, 95% CI 0.06–0.99), higher margin dose (HR 0.23, 95% CI 0.06–0.86) and higher target coverage (HR 0.01, 95% CI 0.00–0.35) were identified as negative predictors, whereas bAVM volume (HR 1.15, 95% CI 1.04–1.27) was a positive predictor of bAVM hemorrhage after SRS. The interaction term between bAVM volume and margin dose was significant and was entered into the multivariable model (Table 2).

**Table 2:** Cox proportional hazard model for predictors of bAVM hemorrhage after SRS.

Characteristic	Univariate			Multivariate		
	HR	95% CI	P value	HR	95% CI	P value
Compact bAVM* (vs. diffuse)	0.47	0.13–1.70	0.248	0.25	0.06–0.99	0.048^†^
Age	0.98	0.93–1.02	0.325	–	–	–
Sex	0.76	0.26–2.16	0.602	0.32	0.10–1.07	0.065
bAVM volume	1.04	1.01–1.07	0.011^†^	1.15	1.04–1.27	0.009^†^
Aneurysm (proximal/intranidal)	1.54	0.34–6.90	0.573	–	–	–
Eloquence	2.43	0.54–10.97	0.243	2.95	0.58–14.97	0.193
Deep location^‡^	NA	NA	NA	–	–	–
Deep venous drainage	2.71	0.91–8.08	0.074	–	–	–
Margin dose	0.42	0.24–0.74	0.003^†^	0.23	0.06–0.86	0.029^†^
Isodose level	1.02	0.81–1.28	0.852	–	–	–
Target coverage^§^	0.06	0.00–0.96	0.046^†^	0.01	0.00–0.35	0.011^†^
bAVM volume* margin dose	NA	NA	NA	1.11	1.03–1.20	0.008^†^

*Compact bAVM is defined as compactness index ≥ 0.63.

^†^P < 0.05.

^‡^No hemorrhage event occurred in deep seated AVM (thalamus, basal ganglia, brainstem).

^§^Target coverage is defined as the percentage of margin dose covered volume within target volume divided by the entire target volume.

The post-SRS hemorrhage rate of each bAVM volume group stratified by bAVM morphology is demonstrated in Table 3. The bAVM size was categorized as < 20 cm^3^, 20–40 cm^3^ and > 40 cm^3^ based on prior clinical experience. Among the diffuse bAVMs, the post-SRS hemorrhage rate was higher for larger bAVMs (1.7 versus 14.9 versus 30.6 hemorrhage per 1000 person-years in bAVM volume < 20 cm^3^ versus 20–40 cm^3^ versus > 40 cm^3^; p = 0.022). The post-SRS hemorrhage rate was not significantly different between bAVM sizes for the compact bAVMs.

**Table 3:** Comparison of post-SRS hemorrhage rates between bAVM volume groups, stratified by bAVM morphology.

bAVM volume	< 20 cm^3^	20–40 cm^3^	> 40 cm^3^	P value
All bAVMs, hemorrhage events/person-years (hemorrhage rate per 1000 person-years)	2/886.6 (2.3)	5/504.4 (9.9)	7/326.6 (21.4)	0.011
bAVM morphology	Hemorrhage events/person-years (hemorrhage rate per 1000 person-years)			
Compact*	1/284.3 (3.5)	0/168.4 (0)	2/163.2 (12.3)	0.492
Diffuse	1/602.3 (1.7)	5/335.9 (14.9)	5/163.4 (30.6)	0.022^†^

*Compact bAVM is defined as compactness index ≥ 0.63.

^†^P < 0.05.

For Spetzler-Martin grade IV–V bAVMs, the post-SRS hemorrhage rate was significantly higher compared with the Spetzler-Martin grade I–III nidi (20.0 versus 3.3 per 1000 person-years; p = 0.001). After stratifying the Spetzler-Martin grade IV–V bAVMs by nidus morphology, we revealed a significantly lower post-SRS hemorrhage rate in the compact bAVMs compared with the diffuse ones (4.8 versus 30.9 per 1000 person-years; p = 0.035), as shown in Table 4.

**Table 4:** Comparison of post-SRS hemorrhage rates between the diffuse and compact bAVMs, stratified by SM grade.

Spetzler-Martin grade	Total				Compact bAVM^‡^				Diffuse bAVM				P value^†^
	n	Hemorrhage event	PY	Hemorrhage rate*	n	Hemorrhage event	PY	Hemorrhage rate*	n	Hemorrhage event	PY	Hemorrhage rate*	
I–II	108	1	633.7	1.6	42	0	184.1	0	66	1	449.5	2.2	0.568
III	86	3	591.6	6.8	37	2	223.9	8.9	49	1	367.7	2.7	0.396
IV–V	69	10	499.0	20.0	32	1	208.0	4.8	37	9	291.1	30.9	0.035^§^

*Per 1000 PY.

^†^Comparison of hemorrhage rates between compact and diffuse bAVMs using Kaplan–Meier analysis and log rank test.

^‡^Compact bAVM is defined as compactness index ≥ 0.63.

^§^P < 0.05.

## Discussion

The hemorrhage rate after SRS for the unruptured bAVMs presented in our study was 8.2 per 1000 person-years, and the rate was 13.6 per 1000 person-years in the latency period. However, stratification of hemorrhage risk after SRS is essential for physicians to further tailor treatment for the unruptured bAVMs. Using the compactness index, we classified the bAVMs into compact and diffuse types. We identified diffuse bAVM, larger bAVM volume and lower margin dose as independent predictors of post-SRS hemorrhage. Among the diffuse bAVMs, nidi larger than 40 cm^3^ bore the highest hemorrhage risk after SRS management (30.6 hemorrhage per 1000 person-years). The post-SRS hemorrhage rate was significantly higher within Spetzler-Martin grade IV–V bAVMs compared to that of grade I–III; moreover, the elevated risk mainly originated from the diffuse type of Spetzler-Martin grade IV–V bAVMs (30.9 hemorrhage per 1000 person-years).

The hemorrhage risk reduction of bAVMs after SRS primarily derives from complete obliteration of the nidi. Ding et al. conducted a hemorrhage risk analysis of 2320 patients who underwent radiosurgery for bAVMs. The authors reported a significantly reduced hemorrhage rate among the obliterated nidi than the patent bAVMs (6.0 and 22.3 hemorrhage per 1000 person-years)^10^. Pollock et al. also reported that radiosurgery failed to reduce the bleeding rate of the non-obliterated bAVMs compared to the untreated natural course in up to 5 years of follow-up^14^. The aim of the current study was not to differentiate the hemorrhage risks between obliterated and non-obliterated bAVMs as patients with only 6 months of follow-up were included. However, a previous study, analyzing 209 unruptured bAVMs with at least 3 years of follow-up, demonstrated that diffuse, larger bAVMs with deep venous drainage have lower probability to achieve complete obliteration after SRS^13^. This could partly explain the independent effect of bAVM morphology (compact versus diffuse) and volume on post-SRS hemorrhage in the present analysis.

In a multicenter study analyzing the risk of bAVM hemorrhage before and after stereotactic radiosurgery, SRS reduced the risk of hemorrhage in Spetzler-Martin grade I–III bAVMs; however, SRS might increase the hemorrhage rate in grade IV–V bAVMs^10^. The current study also revealed unexpectedly high hemorrhage rates within the diffuse bAVMs larger than 40 cm^3^ (30.6 hemorrhage per 1000 person-years) and in the diffuse type grade IV–V bAVMs (30.9 hemorrhage per 1000 person-years). A possible mechanism for either failure to achieve bAVM obliteration or hemorrhage risk increment after SRS was unintentional partial treatment (target miss). Almost half of the incomplete obliteration was associated with inadequate coverage of bAVMs as reported by Pollock et al.^15^ Our data also demonstrated that higher target coverage reduced the risk of post-SRS hemorrhage (see Table 2). In early SRS simulation experiments using theoretical bAVM mathematical model, Lo et al. demonstrated that partial-volume irradiation redistributed blood flow to the bAVM remnants, increasing the pressure gradient which then translated into higher circumferential wall stress and elevated the probability of hemorrhage^16,17^. Massoud et al. used a more complex bAVM model and reported similar results that ruptures occurred after subtotal and low-dose radiosurgery due to hemodynamic shift from the obliterated vessels to the patent ones^18^. In the worst scenario of unintentional partial treatment, the venous side of the bAVM occludes while the arterial feeding side is left inadequately irradiated, predisposing bAVMs to hemorrhage^19–21^. Consistent with the perspective in a radiosurgical research with 509 ARUBA-eligible patients, our finding suggests that partial treatment of bAVMs might destabilize the residual nidi and elevate the hemorrhage rate to a level higher than the natural course^22^. A similar result was also reported by Han et al. in patients with Spetzler-Martin IV–V bAVMs undergoing partial treatment^23^. To summarize, full nidus coverage is substantial for the management of large and diffuse bAVMs. Nevertheless, a larger 12 Gy volume which corresponds to a larger radiation volume predicts the occurrence of permanent RIC in single-session radiosurgery^24^. Facing the dilemma, staged radiosurgery^25,26^, multimodality treatment^27^, or conservative treatment would be reasonable strategies instead of compromising the target coverage for the diffuse and high-grade bAVMs.

Most centers have guidance on dose prescription for bAVMs of different sizes to evade from radiation-induced side effects. The interaction terms between the prescribed margin dose and bAVM volume was significant and added in our multivariable model. The finding that higher margin dose significantly reduced post-SRS hemorrhage corroborates with previous studies^8,10,28,29^ and supports our practice to deliver a sufficient mean dose even when treating large bAVMs^29^. The independent effect of the margin dose could be due to faster obliteration process with higher radiation dose level, which shortened the latency period of bAVMs receiving SRS^30,31^; alternatively, it could reflect that the whole bAVMs were completely covered in a certain level of radiation dose. However, previous studies have demonstrated a positive correlation between the incidence of radiologic or permanent RICs and prescribed dose^13,24,32^. The benefit of dose escalation should be weighed against radiation-induced side effects.

Our cohort only consisted of patients with unruptured bAVMs, thus we adopted the 2.2% annual bleeding rate from published natural history studies to evaluate the protective effect of radiosurgery for bAVM hemorrhage^1,5,6^. The annual hemorrhage rate in our cohort with unruptured bAVMs treated with upfront SRS was 0.8%, which is significantly lower than the simulated natural 2.2% hemorrhage risk based on Kaplan–Meier analysis. Previous studies also reported that SRS may reduce hemorrhage rate of the unruptured bAVMs^22,33–35^. However, certain subgroups of patients could endure higher risk of stroke or death after interventional therapy compared to natural course as hinted by the ARUBA trial and its final report^6,36^. While the natural risk of hemorrhage showed a weak association with the bAVM size^1^, larger bAVM volume has been correlated with increasing hemorrhagic strokes after SRS for the unruptured bAVMs^37,38^. Pollock et al. found that bAVMs > 5.6 cm^3^ had an increased risk of stroke or death after radiosurgery compared with those ≤ 5.6 cm^3^^37^. Similarly, Karlsson et al. showed different risks of morbidity and mortality following SRS between unruptured bAVMs ≤ 5 cm^3^ and > 5 cm^3^^38^. Our data showed that both volumetric and morphologic effects are decisive for post-SRS hemorrhage. Thus, the appropriate size limit for SRS management of unruptured bAVMs should be stratified by the compactness of the nidi. For compact bAVMs, the hemorrhage rate after SRS did not vary with bAVM volume (< 20 cm^3^ versus 20–40 cm^3^ versus > 40 cm^3^) and was consistently below the natural hemorrhage risk. Contrarily, the hemorrhage rate increased with larger bAVM volume among the diffuse bAVMs. Diffuse and large (> 40 cm^3^), or the diffuse Spetzler-Martin IV–V bAVMs might not be good candidates for single-session radiosurgery because the post-SRS hemorrhage rate (30.6 and 30.9 hemorrhagic events per 1000 person-years) could be higher than the natural course. Nevertheless, the treatment decision of unruptured bAVMs is multifactorial. Other SRS management considerations include alleviation of drug-resistant seizure, steal phenomenon or psychological burden with the trade-off of radiation-induced complications.

Our study has several limitations. First, there was no control group for comparison. We could not determine whether the independent factors for post-SRS hemorrhage reflect the natural history of bAVMs or the effects of radiosurgery. However, previous analysis showed that bAVM volume and morphology significantly correlated with complete obliteration after radiosurgery^13^. Since obliteration of bAVMs substantially alter the hemorrhage risk, the predictors found in the current study less likely reflected the natural bleeding tendency, but could serve as a guidance for patient selection for management of unruptured bAVMs. Second, we have no direct measurement of hemodynamic profiles of the bAVMs. Lin et al. utilized quantitative DSA to reveal the correlation between venous outflow restriction and bAVM hemorrhage^21^. Future radiosurgical studies could adopt the less invasive measurements to obtain hemodynamic data. Thirdly, no patient with associated aneurysms, a previously reported predictor for post-SRS hemorrhage^10,39^, experienced hemorrhage in our cohort. The inadequate sample size hampers generalizability of our finding. However, detailed clinical data and the computer treatment plan were required to perform tissue segmentation and relative analysis. The strict requirement increased the difficulty of patient data recruitment.

## Methods

### Patient population and baseline variables

This study was approved by the Taipei Medical University-Joint Institutional Review Board (IRB) and Taipei Veterans General Hospital IRB. Due to retrospective nature of our study and scrambled patient identity, consent to participate was waived. The study followed the Consolidated Standards of Reporting Trials (CONSORT) protocol and was performed in accordance with the Declaration of Helsinki. A total of 1361 patients underwent SRS for bAVMs between January 1993 and December 2020 at two Gamma Knife centers. We included the following patients in this study: (1) patients with unruptured and untreated bAVMs who were treated with single-session SRS; (2) adult patients older than 18 years of age; (3) patients with at least one available clinical or neuroimaging follow-up record. A total of 262 patients satisfied the inclusion criteria.

Patient demographic comprised age, sex and disease presentation. bAVM characteristics consisted of bAVM volume, location, presence of deep venous drainage or proximal/intranidal aneurysms. Deep location was defined as bAVMs at basal ganglia, thalamus and brainstem. Spetzler-Martin grade, modified radiosurgery-based AVM score, and Virginia Radiosurgery AVM Scale score, were calculated for each bAVM. Dosimetry parameters included margin dose, isodose level, maximum dose, target mean dose, and gradient index.

### Follow-up

bAVM hemorrhage after SRS was the outcome of interest in this study, and was defined as any intracranial hemorrhage confirmed by computed tomography (CT) or MRI, regardless of neurological sequelae. The date of hemorrhage, acute and long-term clinical outcome were registered. For patients without bAVM hemorrhage after SRS, their data were censored at the last clinical or image follow-up. Notably, chronic expanding hematomas were not considered as post-SRS hemorrhage^40^. bAVM obliteration was defined as a lack of abnormal flow voids on T2-weighted MRI or arteriovenous shunting on digital subtraction angiography (DSA). For those with residual nidus 3–5 years after initial SRS, repeat SRS was usually recommended. Of them, 4 out of 8 repeated SRS were performed after bAVM hemorrhage and deemed non-causality to the events. Radiation-induced changes (RIC) were defined as perinidal T2-weighted signal hyperintensities. Radiologic RICs, defined as any image sign of RIC, were graded according to Yen’s classification system^41^. Symptomatic RICs were radiologic RIC associated with new or worsening neurological symptoms not related to intracranial hemorrhage. Permanent RICs were defined as unrecoverable symptomatic RICs which still presented at the time of the final follow-up examination.

### Radiosurgery technique

The Leksell Model G frame (Elekta AB) was secured on the skull for patient immobilization under local anesthesia. Digital subtraction angiography (DSA), thin-slice (1–2 mm) MRI, and MRA have been used for all patients since 1993 to facilitate target delineation. SRS was performed with the Leksell Unit Model B from 1993 to 2006, Model 4C from 2006 to 2013, and Perfexion thereafter (Elekta AB). The radiosurgical parameters and dose planning were determined by the treating neurosurgeon in consultation with a medical physicist and radiation oncologist.

### Automated segmentation

Unsupervised machine learning with fuzzy c-means (FCM) clustering was used to differentiate the tissue components within the bAVM irradiation volume on T2-weighted imaging, which provided optimal tissue contrast. The protocol was implemented in the MATLAB environment (MathWorks, Inc.). A high concordance rate has been proved between the results of automated segmentation and the hand-craft contour of senior neurosurgeons^11,12^. The automated process began with interpolating the dose intensity map to the corresponding T2-weighted MRI. The region of interest (ROI) was the region where radiation exposure exceeding the prescribed margin dose (i.e. the prescription isodose volume). The voxels within the ROI were classified as vessel (flow-void), brain tissue (grey), and CSF (white) by the FCM algorithm based on the distribution of T2-weighted MRI signal intensity. The compactness index was previously reported to quantify the morphology (compact/diffuse) of bAVMs^13^. The index was defined as the ratio of the vascular volume to the brain tissue volume, which was equal to the ratio of vascular proportion (%) to the brain tissue proportion (%) within the ROI. In this study, the algorithm was used to categorize the bAVMs into compact or diffuse types.

### Statistical analysis

Data were presented as frequency (percent) for categorical variables and as median (range) for continuous variables. The post-SRS hemorrhage rate was calculated with the number of hemorrhagic events divided by the person years from the SRS date to the latest follow-up. In order to dichotomize the continuous variables, we generated a receiver-operating characteristics (ROC) curve and calculated Youden indices to determine the cutoff for compactness index. Cox proportional hazard model with backward stepwise selection using 0.157 as stopping criterion^42^ was used to determine the association between post-SRS hemorrhage and patient demographics, bAVM characteristics, and dosimetry parameters. No collinearity between variables was noted. Potential interactions between variables were evaluated by using interaction terms, and a significant interaction term was added into the multivariable model. We simulated natural actuarial hemorrhage rates using an annual bleeding rate of 2.2%, and compared them to the actuarial hemorrhage rates of the diffuse and compact types of bAVMs with Kaplan–Meier analysis and log rank test. A p value < 0.05 was considered statistically significant, and all tests were 2-tailed. All analyses were performed using SPSS version 22 for Windows (IBM Corp.).

## Conclusion

bAVM morphology, volume and margin dose were significant predictors for post-SRS hemorrhage of the unruptured bAVMs. Further stratification of the post-SRS hemorrhage risk showed that diffuse and large bAVMs (> 40 cm^3^), or diffuse type Spetzler-Martin IV–V bAVMs might be inappropriate candidates for single-session radiosurgery where the hemorrhage rate exceeded 2.2% per year. The finding of this study could help patient selection for the management of the unruptured bAVMs.

### Supplementary Information

Supplementary Information.

## Data Availability

The datasets generated during and/or analyzed during the current study are available from the corresponding author on reasonable request.

## Abbreviations

ARUBA: A randomized trial of unruptured brain arteriovenous malformations
bAVM: Brain arteriovenous malformation
CT: Computed tomography
DSA: Digital subtraction angiography
FCM: Fuzzy c-means
MRI: Magnetic resonance images
RIC: Radiation-induced change
ROI: Region of interest
SRS: Stereotactic radiosurgery
