# Supplementary material for: Vascular compactness of unruptured brain arteriovenous malformation predicts risk of hemorrhage after stereotactic radiosurgery
Source: Sci Rep. 2024 Feb 18;14:4011. doi: 10.1038/s41598-024-54369-2 (PMC10874940; doi:10.1038/s41598-024-54369-2)

| **Supplementary Table 1.** Clinical details of Hemorrhagic events after SRS | | | | |
| --- | --- | --- | --- | --- |
| bAVM location | Time-to-hemorrhage (months) | Symptoms associated with post-SRS bAVM hemorrhage | | |
|  |  | Acute | Long-term | |
| Left cerebellopontine angle | 5.2 | Acute headache with nausea and vomiting | Symptom subsided | |
| Left frontal lobe | 8.9 | Right leg twitching | Symptom subsided | |
| Left occipital lobe | 21.3 | Acute headache with nausea and vomiting | Symptom subsided | |
| Left cerebellum | 21.8 | Altered mental status, E2V4M6 | CNS infection (mortality) | |
| Left frontal lobe | 22.8 | Altered mental status, E1V2M5 | Right hemiparesis and monotonous speech | |
| Left frontal lobe | 29.9 | Increased seizure frequency | Seizure controlled by anticonvulsants | |
| Right parieto-temporal lobe | 31.7 | Left limbs weakness with acute headache | Left hemiplegia and hemiparesthesia | |
| Left frontal lobe | 43.0 | Acute headache | Symptom subsided | |
| Right temporal lobe | 43.8 | Altered mental status, E1V1M4 with the right pupil dilatation | Left hemiparesis, dysphagia and aphasia | |
| Left occipital lobe | 48.8 | Transient left limbs weakness and headache | Right visual field defect | |
| Left fronto-temporal lobe | 61.6 | Altered mental status, E1V1M1 | Right hemiplegia, drop foot and motor aphasia | |
| Left frontal lobe | 87.4 | Altered mental status, E1VtM4 | Right visual field defect and right lower limb hemiparesis | |
| Right parietal lobe | 123.2 | Left central type facial palsy with drooling and left lower limb muscle weakness | Left lower limb hemiparesis | |
| Left temporal lobe (hippocampus) | 185.7 | Acute headache and dizziness | Left hemiparesis | |

| **Supplementary Table 2.** Comparison of post-SRS hemorrhage rates between the diffuse and compact bAVMs, stratified by obliteration status | | | | | | | | | | | | | |
| --- | --- | --- | --- | --- | --- | --- | --- | --- | --- | --- | --- | --- | --- |
| Obliteration status | Total | | | | Compact bAVM^‡^ | | | | Diffuse bAVM | | | | P value^†^ |
|  | n | Hemorrhage event | PY | Hemorrhage rate* | n | Hemorrhage event | PY | Hemorrhage rate* | n | Hemorrhage event | PY | Hemorrhage rate* |  |
| Completely obliterated | 196 | 9 | 1356.6 | 6.6 | 76 | 3 | 480.8 | 6.2 | 120 | 6 | 875.8 | 6.9 | 0.818 |
| Patent | 67 | 5 | 284.6 | 17.6 | 35 | 0 | 113.7 | 0 | 32 | 5 | 170.9 | 29.3 | 0.025^§^ |
| *per 1000 PY  ^†^Comparison of hemorrhage rates between compact and diffuse bAVMs using Cox proportional hazards model  ^‡^Compact bAVM is defined as compactness index ≥ 0.63  ^§^P<0.05 | | | | | | | | | | | | | |

**Supplementary Figure 1.** A 23-year-old male with a left frontal lobe bAVM presented as seizure. The bAVM volume and the prescribed margin dose were 49.7 cm^3^ and 16.5 Gy. **A and B:** Figures demonstrate the segmentation results within in the prescription isodose volume. (*Red* indicates the vessel component, *green* indicates the brain parenchyma, and *blue* indicates the CSF.) The compactness index was 0.42, which allocated the bAVM into diffuse morphological type. **C and D:** Lateral and anteroposterior angiography of the left vertebral artery. **E-G:** The follow-up MRI at 12, 44 and 68 months after SRS showed a small residual nidus. **H:** The patient suffered a hemorrhage event 86 months after SRS as demonstrated on the non-contrast CT.


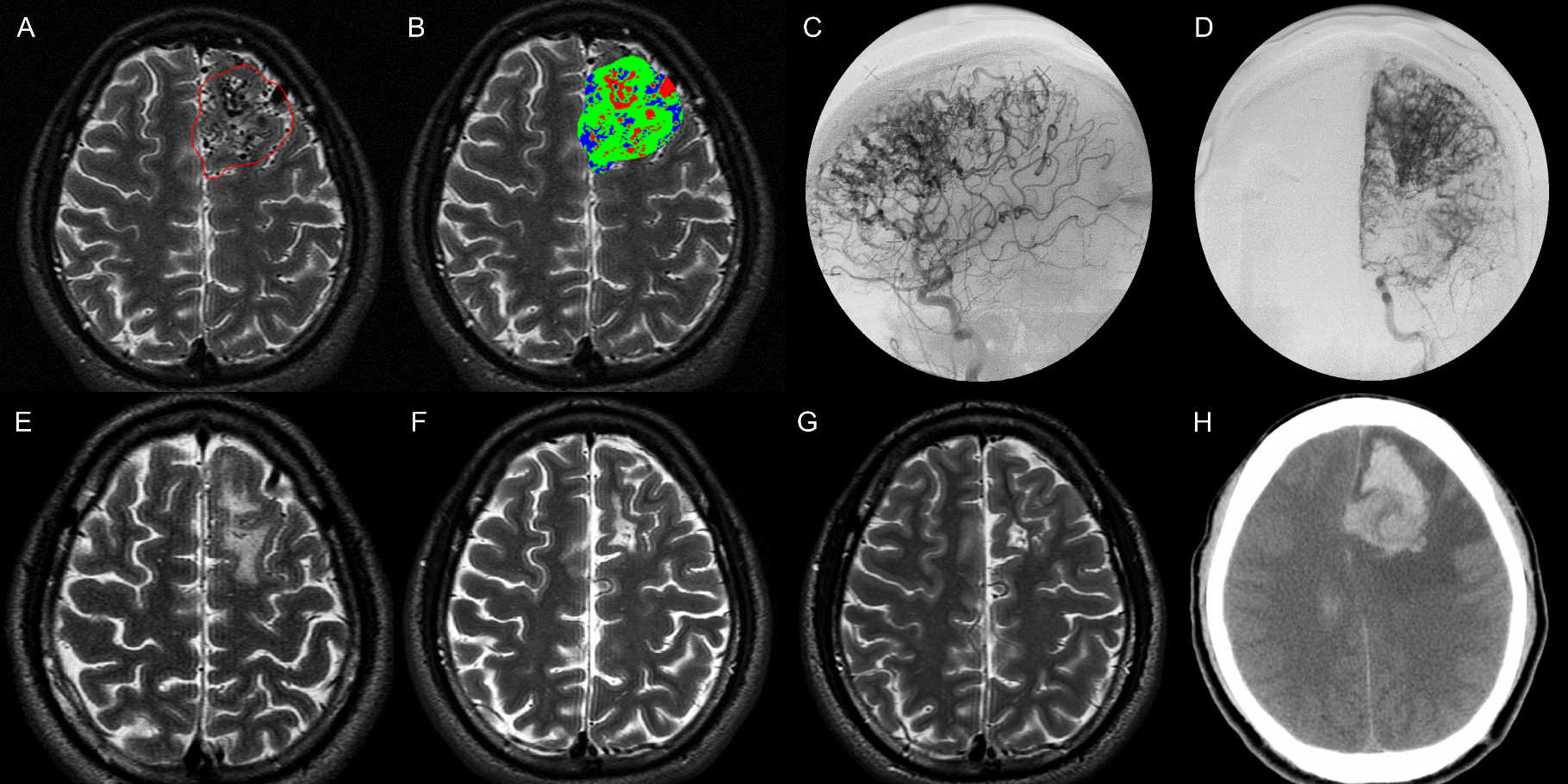

Supplement: Supplementary file 1 — Supplementary Information. [file 41598_2024_54369_MOESM1_ESM.docx]
